# Taxonomic notes on Babinskaiidae from the Cretaceous Burmese amber, with the description of a new species (Insecta, Neuroptera)

**DOI:** 10.3897/zookeys.748.24198

**Published:** 2018-04-04

**Authors:** Jiahui Hu, Xiumei Lu, Bo Wang, Xingyue Liu

**Affiliations:** 1 Department of Entomology, China Agricultural University, Beijing 100193, China; 2 State Key Laboratory of Palaeobiology and Stratigraphy, Nanjing Institute of Geology and Palaeontology, Chinese Academy of Sciences, Nanjing, China

**Keywords:** Mesozoic, Myrmeleontoidea, Neuropterida, phylogeny, taxonomy

## Abstract

Babinskaiidae is an extinct lacewing family of the superfamily Myrmeleontoidea. Hitherto, nine species of seven genera are described from the Lower and mid-Cretaceous. Here a new species of Babinskaiidae is described from Cretaceous Burmese amber, namely *Parababinskaia makarkini* **sp. n.** The new species possesses an A2 vein in the hind wing, suggesting that the loss of this vein might not be an autapomorphy of Babinskaiidae. The female of *Electrobabinskaia burmana* Lu, Zhang & Liu, 2017 is also described for the first time based on two specimens with their abdomens perfectly preserved, exhibiting a specialised sternum VI with paired elongate projections. A brief discussion of female genital characters is provided, which may increase our understanding of the morphology and phylogenetic position of Babinskaiidae.

## Introduction

The extinct lacewing family Babinskaiidae, belongs to the superfamily Myrmeleontoidea, and is recently considered to form an epifamily Nymphidoidae together with Nymphidae (Makarkin et al. 2017). Adults of Babinskaiidae can be characterised by long filiform antennae, narrowly elongated wings, with features such as trichosors, and presectorial cross veins present in both wings, and absence of forewing oblique vein (i.e., the base of MP2).

Hitherto, Babinskaiidae were only recorded in the Lower Cretaceous of Brazil (Crato Formation) and Russia (Zaza Formation), and the mid-Cretaceous of Myanmar (Martins-Neto and Vulcano 1989a, b; Ponomarenko 1992; Martins-Neto 1997; Lu et al. 2017; Makarkin et al. 2017). Currently, the family contains nine species assigned in seven genera, i.e., *Baisonelia* Ponomarenko, 1992 from the Lower Cretaceous of Russia; *Babinskaia* Martins-Neto & Vulcano, 1989, *Neliana* Martins-Neto, 1992, *Parababinskaia* Makarkin, Heads & Wedmann, 2017 from the Lower Cretaceous of Brazil; and *Burmobabinskaia* Lu, Zhang & Liu, 2017, *Electrobabinskaia* Lu, Zhang & Liu, 2017, and *Pseudobabinskaia* Makarkin, Heads & Wedmann, 2017 from the mid-Cretaceous of Myanmar. However, many of these species are from compression fossils, and some of them are known only from wing fragments (e.g., *Baisonelia vitimica* Ponomarenko, 1992 and *Neliana impolluta* Martins-Neto, 1997, each with only a hind wing preserved). Recent discovery of Babinskaiidae in Burmese amber provides important evidence to understand the morphology, taxonomy, and phylogenetic status of this family owing to the well-preserved specimens (Lu et al. 2017). Nevertheless, known Burmese amber specimens of Babinskaiidae are still scarce.

In this paper, with examination of more specimens of Babinskaiidae from the Burmese amber, a new species of *Parababinskaia* is reported based on two specimens with both the male and the female described, and the female of *Electrobabinskaia burmana* Lu, Zhang & Liu, 2017 is also described for the first time. A comparative study on the female genital morphology of Babinskaiidae is presented.

## Materials and methods

The amber samples described are from the Hukwang Valley in Tanai Township, Myikyina District of Kachin State, Myanmar (Kania et al. 2015). The age of this deposit has been investigated and dated to be 98.8±0.6 million years by U-Pb dating of zircons from the volcanoclastic matrix of the amber (Shi et al. 2012).

The specimens are deposited in the Nanjing Institute of Geology and Palaeontology, Chinese Academy of Sciences, Nanjing, China, while a paratype of the new species herein described is currently housed in the Entomological Museum, China Agricultural University (**CAU**), Beijing, and will eventually be deposited in the Collection of Xiao Jia in the Century Amber Museum (**CAM**), Shenzhen.

Photographs and drawing were taken and made using a Zeiss SteREO Discovery V12 microscope system. The figures were prepared with Adobe Photoshop CS6. Terminology of wing venation generally follows Aspöck et al. (1980) and Martins-Neto (2000). Breitkreuz et al. (2017) presented an alternative interpretation on the homology of wing venation in Neuropterida based on vein tracheation. The corresponding abbreviations of the veins based on the nomenclature in Breitkreuz et al. (2017) are given below in the parentheses for comparison. These two venation terminologies differ from each other mainly in homology interpretation and definition of MA, i.e., whether MA is considered to be fused with RP at the wing base. The venation terminology used for Babinskaiidae in Makarkin et al. (2017) is in generally similar to that of Breitkreuz et al. (2017). Terminology of genitalia follows Aspöck and Aspöck (2008).

Abbreviations used for wing veins are:

**A (A)** anal vein;

**C (C)** costa;

**Cu (Cu)** cubitus;

**CuA (CuA)** cubitus anterior;

**CuP (CuP)** cubitus posterior;

**M (M)** media;

**MA (RP1)** media anterior;

**MP (MA+MP)** media posterior;

**R (R)** radius;

**RA (RA)** radius anterior;

**RP (RP)** radius posterior;

**ScP (Sc)** subcosta posterior;

**ps** presectorial crossveins (i.e., r-mp crossveins).

## Systematic palaeontology

### Class Insecta Linnaeus, 1758

#### Order Neuroptera Linnaeus, 1758

##### Superfamily Myrmeleontoidea Latreille, 1802

###### Epifamily Nymphidoidae Rambur, 1842

####### Family Babinskaiidae Martins-Neto & Vulcano, 1989

######## Genus Parababinskaia

Taxon classificationAnimaliaNeuropteraBabinskaiidae

Makarkin, Heads & Wedmann, 2017

Figs 1 , 2 , 3 , 4

Parababinskaia Makarkin, Heads & Wedmann, 2017: 153. Type species: Parababinskaia elegans Makarkin, Heads & Wedmann, 2017: 153 (original designation).

######### Revised diagnosis.

Forewing: Narrowly elongate, slightly broadened distally, with four or five presectorial cross veins. RP+MA originating proximal of the termination of CuP, RP with five branches, most of which are simple. Six cross veins present between RA and RP. MP pectinately branched about at distal 1/5. CuA pectinately branched. A1 bifurcated. A2 and A3 present, and not fused with each other. A short outer gradate series of cross veins present. Hind wing: Slightly narrower than forewing. Three or four presectorial cross veins present. RP+MA originating almost at same level with termination of CuA. RP with four or five branches, posterior three branches of which are simple. Four to seven cross veins present between RA and RP. MP1 pectinately branched approx. at distal 1/5. MP2 pectinately branched nearly at its midpoint. CuP and A1 proximally fused. A2 present. Female abdominal segment VI without projections on sternum.

######## Parababinskaia makarkini sp. n.

Taxon classificationAnimaliaNeuropteraBabinskaiidae

http://zoobank.org/4B1E6677-C7E3-4ADC-968F-AE3988A8CC10

Figs 1 , 2 , 3 , 4

######### Diagnosis.

Many CuA branches in forewing bearing small marginal fork. Hind wing with four or five cross veins between RA and RP, and with eight branches of MP2.

######### Description.

**Figure 1.**
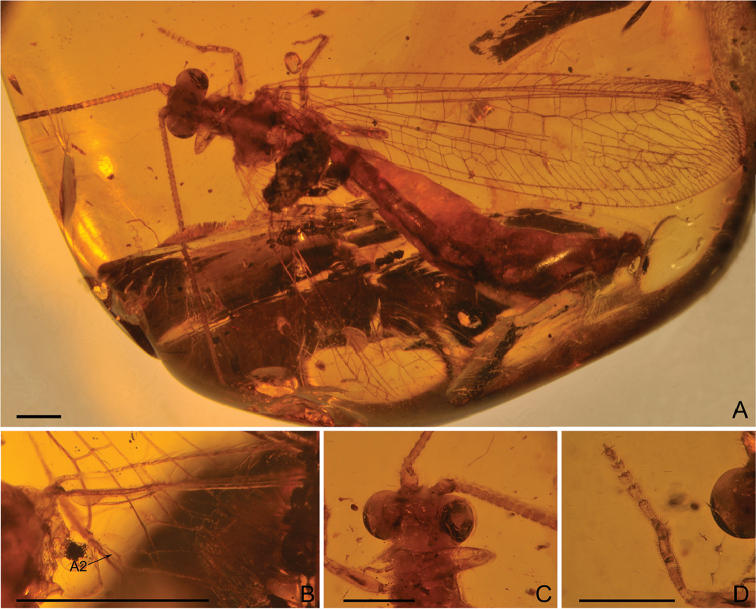
*Parababinskaia makarkini* sp. n., holotype male. **A** Habitus photograph, dorsal view **B** Photograph of left wing base **C** Photograph of head, ventral view **D** Photograph of tarsus. Scale bars: 1.0 mm.

***Male*** (Fig. 1A). *Body* length 11.20 mm; head 0.90 mm long and 1.70 mm wide; antenna length 6.34 mm; forewing 11.11 mm long and 2.90 mm wide; hind wing 9.37 mm long and 2.52 mm wide. Abdomen length 7.64 mm.

*Head* with vertex with a pair of domed regions (Fig. 1C). Compound eyes large, semi-globular. Antenna filiform, with dense short setae; scape much wider and longer than pedicel; flagellum with 49 flagellomeres, each flagellomere much longer but narrower than pedicel.

*Prothorax* slightly longer but much narrower than head, laterally with some long hairs. Meso- and metathorax robust. Wings in general narrowly elongated, transparent, and immaculate.

*Forewing* with single trichosors between veins along distal margin; multiple trichosors (up to seven) between veins along costal and posterior margins. Costal space about three times as wide as subcostal space, but much narrower than radial space, with 18 simple veinlets on proximal 3/4 and 16 marginally forked, more inclined veinlets on distal 1/4; only one subcostal cross vein (1scp-r) present near the wing base. Four presectorial cross veins present. Origin of RP+MA slightly proximad termination of CuP. MA diverging from RP much distad separating point of RA and RP+MA; RP with five branches, and only anterior-most one bearing a small marginal fork. Six cross veins present in radial space. MA with a small marginal fork. MP long and straight, pectinately branched about at its distal 1/5, and all branches with a small marginal fork. A short outer gradate series cross veins present. Eleven crossveins present between MP and CuA. CuA and CuP diverging near wing base. CuA pectinately branched and slightly zig-zagged distally, with eight branches, most of which bear a small marginal fork. CuP pectinately branched, with six simple branches. Eight cua-cup cross veins present. A1 distally bifurcated. Two cup-a1 cross veins present. A2 and A3 short and simple, not fused with each other.

*Hind wing*: Slightly narrower than forewing. Trichosors as in forewing. Costal space nearly two times as wide as subcostal space, with 14 simple veinlets on proximal 3/4 while with 14 marginally forked veinlets on distal 1/4. Subcostal crossvein absent. Three or four presectorial crossveins present. RP+MA originating nearly at same level of termination of CuA. Four crossveins present in radial space. MP1 and MP2 diverging near wing base; MP1 straight and long, pectinately branched approx. at its distal 1/5, and all branches bearing a small marginal fork; MP2 slightly zig-zagged distally, with eight pectinate branches (anterior three of them with a small marginal fork). Eight or nine intermedia cross veins present. CuA short, with five simple branches. CuP and A1 proximally fused, CuA distally strongly zig-zagged. A2 present, short and simple, slightly curved posteriad (Fig. 1B). An oblique a1-a2 crossvein present. Jugal lobe present.

**Figure 2.**
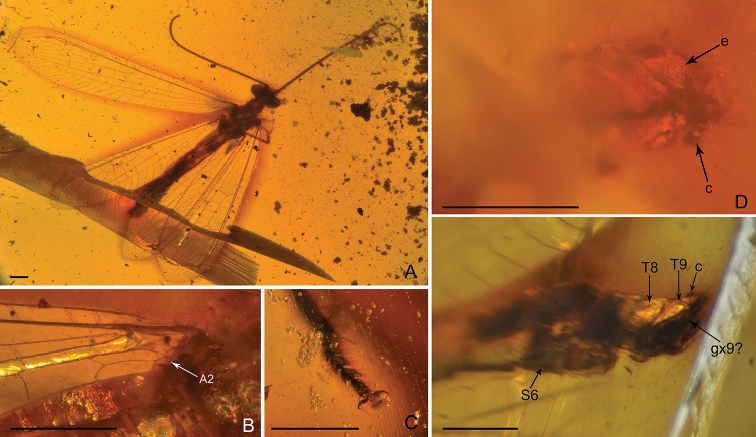
*Parababinskaia makarkini* sp. n., paratype female. **A** Habitus photograph, dorsal view **B** Photograph of left hind wing base, dorsal view **C** Photograph of tarsus **D** Photograph of female genitalia, dorsal view **E**. Photograph of female genitalia, ventral view. Abbreviations: T: tergum; S: sternum; c: callus cercus; e: ectoproct; gx: gonocoxite. Scale bars: 1.0 mm.

*Legs* slender, with dense short setae; specialised setae absent (Figs 1D, 2C). Tarsus 5-segmented; tarsomere I slender, slightly longer than each of the rest tarsomeres; tarsomeres II-IV slightly wider than tarsomere I and feebly tapering on distal-lateral corners; tarsomere V ovoid. Pretarsal claws equal in length and shape, shorter than tarsomere V, without additional teeth. Arolium present, slightly shorter than pretarsal claw.

*Abdomen* slenderly elongate, with segments IV–VI slightly broadened.

*Male genitalia* (Fig. 4A–D): Tergum IX short; sternum IX invisible, probably rather small. Ectoprocts paired, broadly ovoid, with large callus cerci. A seemingly paired, darkly coloured (probably strongly sclerotised) sclerites (putative gonocoxite IX) present beneath ectoprocts and extending well beyond tergum IX.

***Female*** (Fig. 2A). *Body* length 10.68 mm; head 0.86 mm long and 1.32 mm wide; antenna length 8.55 mm; left forewing 13.05 mm long and 2.85 mm wide; left hind wing 10.37 mm long and 2.27 mm wide; right forewing (probably distorted) 10.09 mm long and 3.51 mm wide; right hind wing (probably distorted) 9.42 mm long and 2.75 mm wide; abdomen 7.14 mm long.

**Figure 3.**
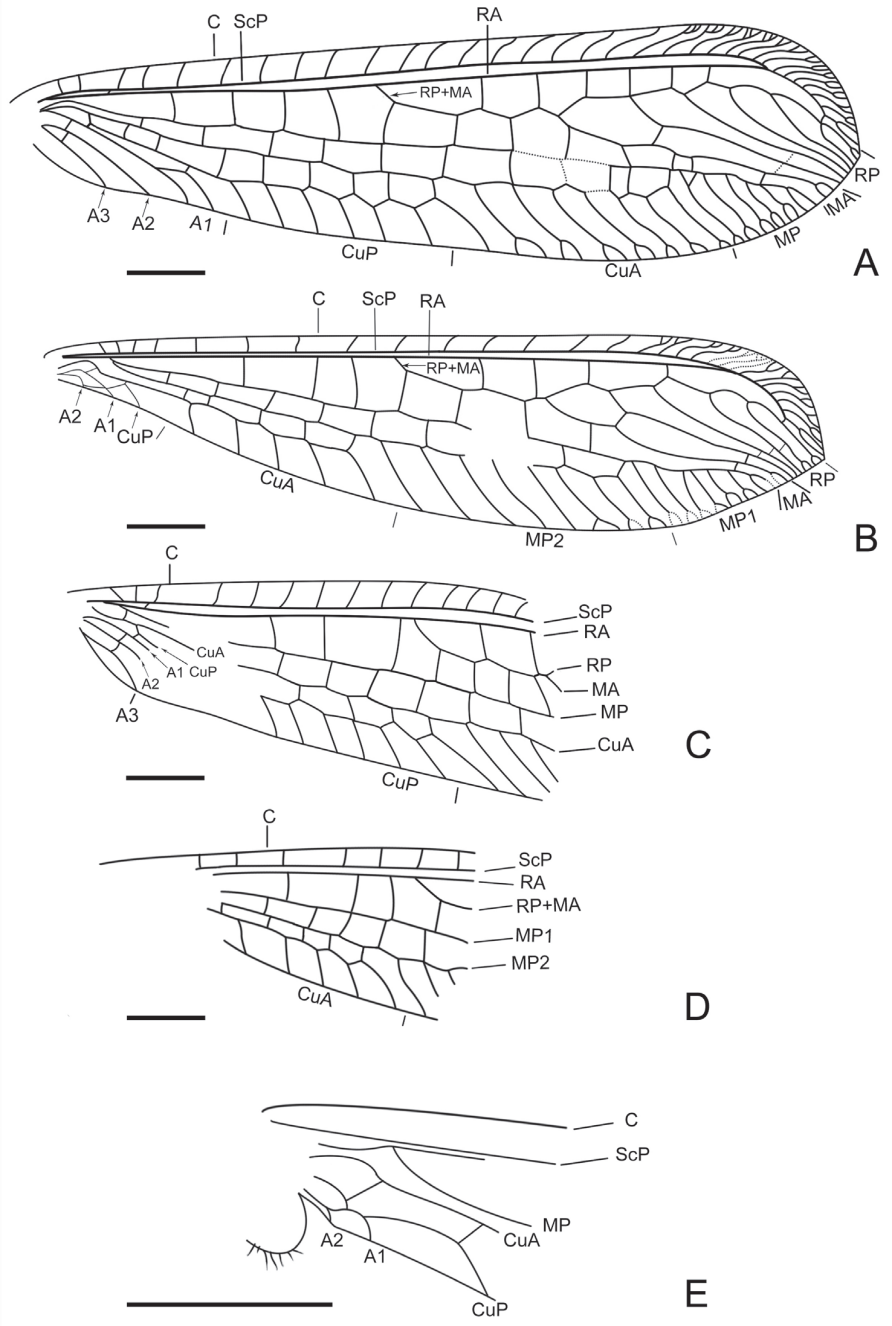
Wing venation of *Parababinskaia makarkini* sp. n., male. **A** Line drawing of right forewing **B** Line drawing of right hind wing **C** Line drawing of left forewing **D** Line drawing of left hind wing **E** Basal part of left hind wing. Scale bars: 1.0 mm.

**Figure 4.**
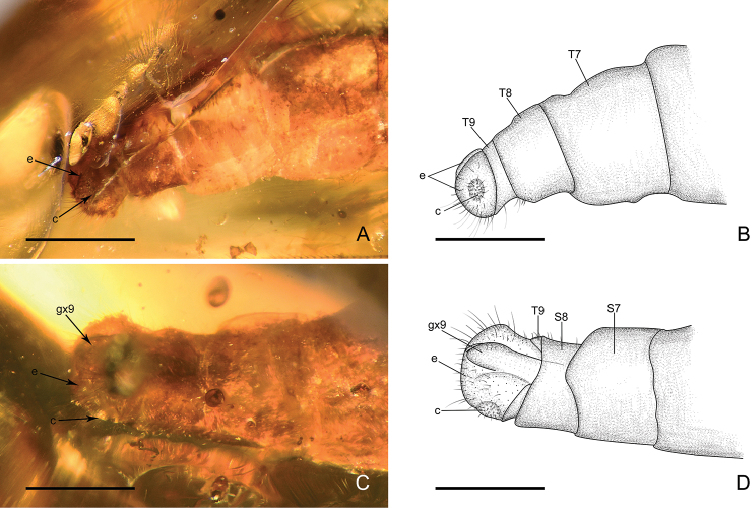
Genitalia of *Parababinskaia makarkini* sp. n., male. **A** Photograph of genitalia, lateral view **B** Drawing of genitalia, lateral view **C** Photograph of genitalia, ventral view **D** Drawing of genitalia, ventral view. Abbreviations: T: tergum; S: sternum; c: callus cercus; e: ectoproct; gx: gonocoxite. Scale bars: 1.0 mm.

External morphology of female almost same as male. Antenna slightly longer, with 59 flagellomeres.

*Forewing*: Five presectorial cross veins present. MP with six pectinate branches, almost all bearing marginal fork. Fourteen cross veins present between MP and CuA. Six cua-cup crossveins present. Only one cup-a1 cross vein present.

*Hind wing*: Four presectorial cross veins present. Five cross veins present on radial space. RP with four branches. MP1 with ten pectinate branches; MP2 with nine simple branches; seven cross veins present between MP1 and MP2. CuA with six simple branches. A2 present (Fig. 2B).

*Abdomen* slender and elongated, with segments V–VII slightly broadened. Segment VI nearly rectangular, posteriorly without specialised projections.

*Female genitalia* (Fig. 2D–E): Tergum VIII broad, nearly rectangular in dorsal view, subtriangular in lateral view. Tergum IX arcuate in dorsal view, notably smaller than tergum VIII. A pair of putative gonocoxite IX present. Ectoprocts paired, cone-like, each with a short and slender projection posteriad. Callus cerci present, large.

######### Type material.

Holotype: NIGP197965: Amber piece preserving a nearly complete male of *Parababinskaia makarkini* sp. n., it is polished in the form of arched pentagon cabochon, clear and transparent, with length × width about 24.18 × 21.44 mm, height 7.76 mm. Paratype: CAM BA-0012: amber piece preserving a complete female of *P. makarkini* sp. n. and a coleopteran larva, it is polished in the form of flattened rectangular cabochon, clear and transparent, with length × width about 3.66 × 23.92 mm, height 6.95 mm.

######### Etymology.

The new species is dedicated to Dr. Vladimir N. Makarkin for his great contributions on the taxonomy of fossil lacewings.

######### Remarks.

The new species is placed in *Parababinskaia* based on the similar number of presectorial crossveins (four or five in the forewing, and three or four in the hind wing), the presence of hind wing outer gradate series of crossveins, and the similar configuration of hind wing CuP, in comparison with the type species of *Parababinskaia*, i.e., *P. elegans*. However, the new species can be distinguished from *P. elegans* by the forewing CuA with most branches marginally forked (most branches of forewing CuA simple in *P. elegans*), the presence of four or five hind wing radial cross veins (six or seven in *P. elegans*), and the presence of eight branches of hind wing MP2 (11 or 12 in *P. elegans*). The new species apparently differs from the other Burmese amber babinskaiids by the bifurcated forewing A1.

The association between the male and female of the new species is based on the similar body size, the generally same wing venation, and the similar tarsi, with tarsomeres II–IV feebly tapering on distal-lateral corners.

######## Genus Electrobabinskaia

Taxon classificationAnimaliaNeuropteraBabinskaiidae

Lu, Zhang & Liu, 2017

Figs 5 , 6

Electrobabinskaia Lu, Zhang & Liu, 2017: 20 Type species: Electrobabinskaia burmana Lu, Zhang & Liu, 2017: 20 (original designation).

######### Revised diagnosis.

Forewing: RP+MA originated from R nearly at proximal 1/3 of wing. Five presectorial crossveins present. RP densely branched with 6–8 branches, most of which bears a marginal fork. CuA branched on distal half, with 9–10 branches, most of which bears a marginal fork; CuP distally zig-zagged, with 6–8 branches, most of which are simple. A1 simple, proximally approximating CuP stem; A2 and A3 simple. Hind wing: Slightly narrower than forewing, proximal part of wing distinctly narrowed, and wing apex acutely pointed and slightly bended posteriad. Three presectorial cross veins present. RP densely branched, most of which bears a marginal fork. MP1 pectinately branched into 5–6 branches; MP2 with 10 branches, most of them are simple. CuA short, with 5–6 simple branches; CuP and A1 possibly fused into CuP+?A1, short and simple. A2 present. Tarsomeres II–IV semilunar, and gradually shortened, tarsomere V ovoid; arolium present. Abdominal segment VI of female with a pair of long digitiform sternal projections.

######### Description.

***Female.*** *Body* length 9.83 mm; head 0.60 mm long and 1.32 mm wide; antenna length 6.60 mm; forewing 10.20 mm long and 3.13 mm wide; hind wing 9.51 mm long and 2.34 mm wide; prothorax 0.58 mm long and 0.62 mm wide; mesothorax 1.27 mm long and 1.59 mm wide; metathorax 0.67 mm long and 1.27 mm wide; abdomen length 6.71 mm.

**Figure 5.**
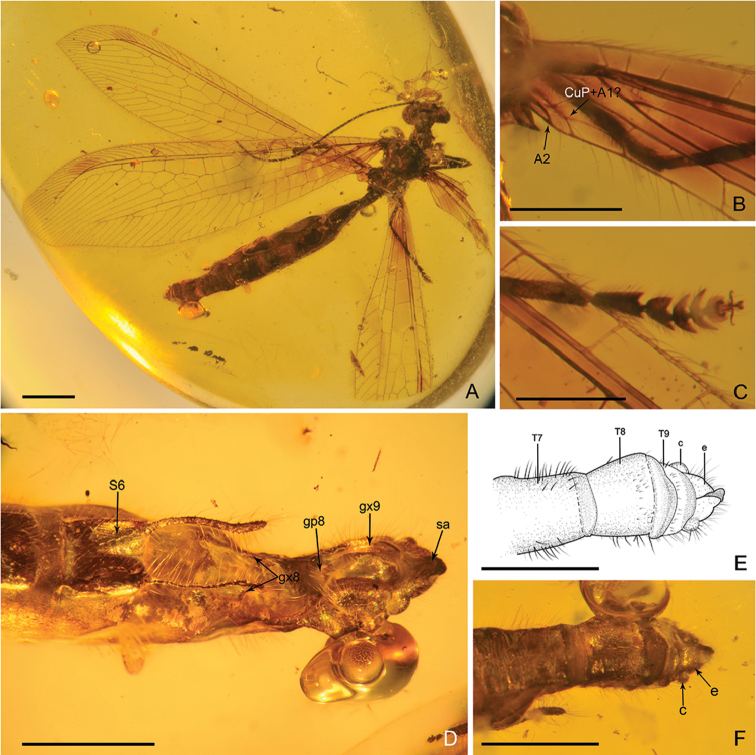
*Electrobabinskaia burmana* Lu, Zhang & Liu, 2017, female. **A** Habitus photograph, dorsal view **B** Photograph of right wing base **C** Photograph of tarsus **D** Photograph of female genitalia, ventral view **E** Line drawing of female genitalia, dorsal view **F** Photograph of female genitalia, dorsal view. Abbreviations: T: tergum; S: sternum; c: callus cercus; e: ectoproct; gp: gonapophysis; gx: gonocoxite; sa: subanale. Scale bars: 0.5 mm (**C**); 1.0 mm (**A–B, D–F**).

External morphology similar to male. But a simple hind wing A2 present (Fig. 5B).

*Abdominal* segment VI with specialised sternum VI. Sternum VI subquadrate, posteriorly concaved, laterally with a pair of long digitiform projections, which are slightly longer than major part of sternum VI, slightly sinuated, bearing long setae.

**Figure 6.**
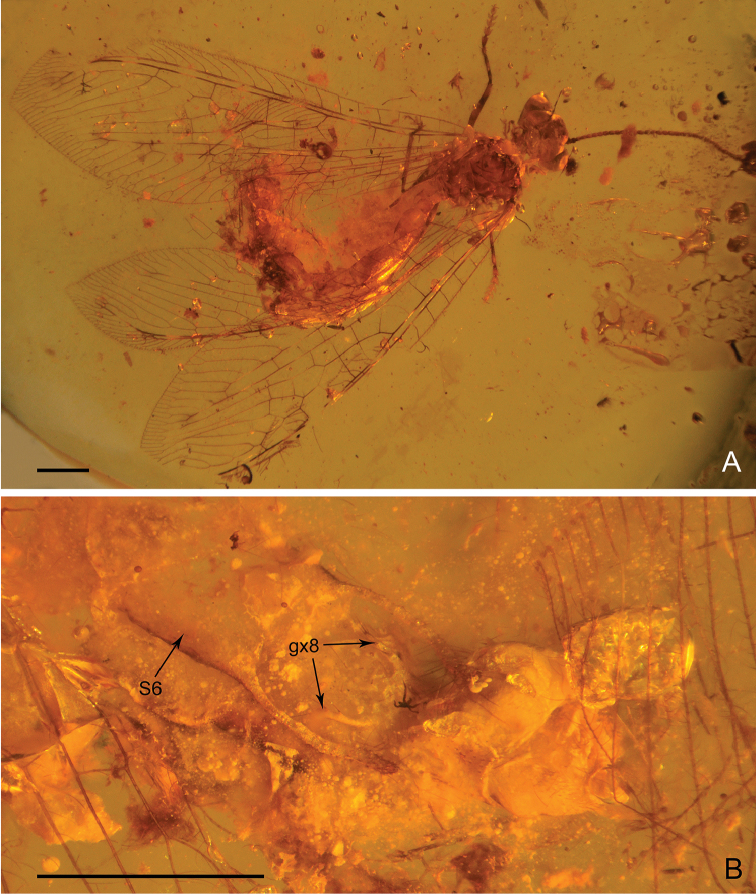
*Electrobabinskaia burmana* Lu, Zhang & Liu, 2017, female. **A** Habitus photograph, dorsal view **B** Photograph of abdomen, ventral view. Abbreviations: S: sternum; gx: gonocoxite. Scale bars: 1.0 mm.

*Female genitalia* (Figs 5D–F, 6B): Tergum VIII subquadrate; gonocoxite VIII paired, present as narrow ridges; putative gonapophysis VIII present, nearly semicircular. Tergum IX in dorsal view arcuate, distinctly enlarged ventrally; a pair of gonocoxite IX present, broadly valvate. Ectoprocts paired, in dorsal view subtriangular, with large callus cerci; a digitiform sclerite present ventral ectoprocts, putatively subanale.

**Figure 7.**
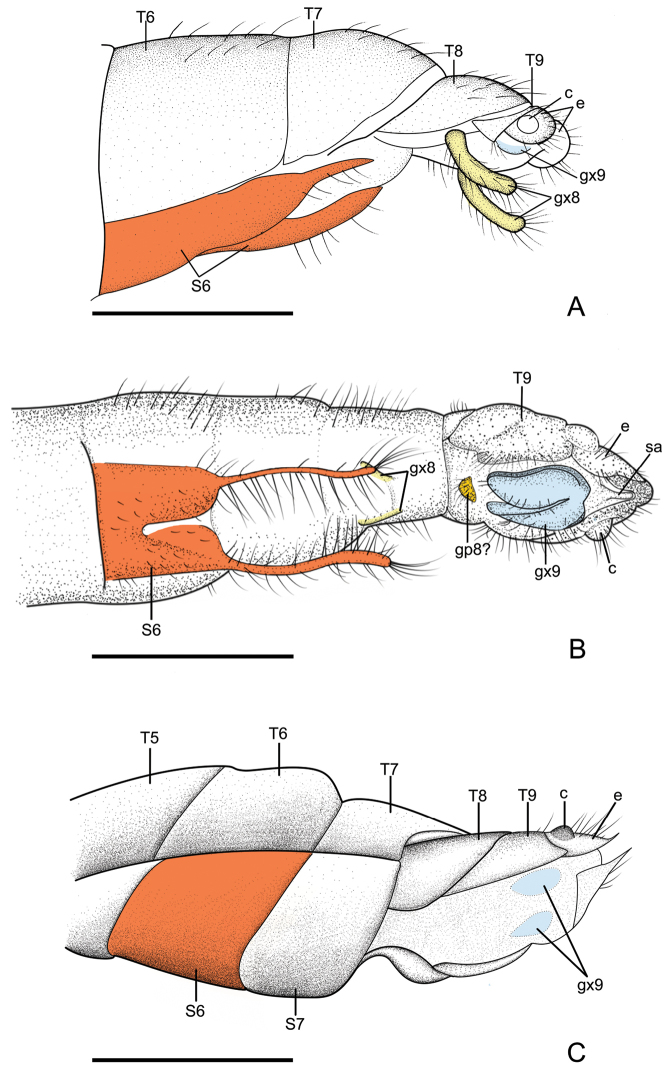
Female genitalia of babinskaiid species from the Burmese amber. **A** Female genitalia of *Pseudobabinskaia martinsnetoi* (Lu, Zhang & Liu) **B** Female genitalia of *Electrobabinskaia burmana* Lu, Zhang & Liu **C** Female genitalia of *Parababinskaia makarkini* sp. n. Abbreviations: T: tergum; S: sternum; c: callus cercus; e: ectoproct; gp: gonapophysis; gx: gonocoxite; sa: subanale. Scale bars: 1.0 mm.

######### Additional material.

NIGP197966: Amber piece preserving a complete female of *E. burmana* and a midge; it is polished in the form of a flattened elliptical cabochon, clear and transparent, with length × width 18.85 × 21.44 mm, height 7.76 mm. NIGP197967: Amber piece preserving a complete female of *E. burmana*; it is polished in the form of a flattened rectangular cabochon, clear and transparent, with length × width 18.94 × 14.34 mm, height 3.31 mm.

######### Remarks.

Association between male and female of *E. burmana* is based on the similar body length (approximately 10 mm), the nearly identical wing venations, and the similar tarsi with semilune tarsomeres II-IV.

## Discussion

The present new findings on the Burmese amber babinskaiids provide important information to further understand the morphology and systematics of Babinskaiidae. Makarkin et al. (2017) outlined four apomorphic wing characters to define Babinskaiidae, including the distal origin of RP+MA, the presence of presectorial cross veins in both wings, the single forewing MP, and the reduction of hind wing A2 and A3. The former three characters may be verified as the synapomorphies of Babinskaiidae although the distal origin of RP+MA and the presence of presectorial cross veins are also present in some lineages of Myrmeleontidae and Ascalaphidae. The long hypostigmal cell is another apomorphic character of Babinskaiidae mentioned in Lu et al. (2017), while it is also present in Nymphidae, Nemopteridae, Palaeoleontidae, and Myrmeleontidae. However, the hind wing A2, possible with A3 merged, is present in *P. makarkini* sp. n. and *E. burmana*, indicating that the reduction of A2 and A3 may not be the autapomorphy of Babinskaiidae as proposed by Makarkin et al. (2017).

The specialised sternum of abdominal segment VI in the female of *E. burmana* is remarkable. In the female of this species there is a pair of long digitiform projections, while such projections are not developed in the conspecific males. In light of the absence of these projections in males, this feature probably functions during courtship or mating although it does not belong to the genital segments. Notably, such modification of abdominal segment VI has never been found in Neuroptera. Previously reported sexually dimorphic features on pregenital segments of abdomen in Neuroptera are only known in males, such as the eversible sacs in some species of Nevrorthidae, Osmylidae and Mantispidae, and the hair pencils in some species of Myrmeleontidae, presumably being involved with chemical communication between sexes (New 1989). However, females of some species of Corydalidae (Megaloptera), e.g., *Protohermes differentialis* (Yang & Yang, 1986), display unusual features on ventral sclerites of abdomen, such as paired long projections on gonocoxites VIII (see Liu and Yang 2006: figs 16–17). In Neuroptera, females of Osmylidae have complex modifications of gonocoxites VIII and gonapophysis IX for grasping males during copulation (Martins et al. 2016). While the above female traits in Babinskaiidae and other groups of Neuropterida are clearly not homologous we cannot exclude that they may similarly function considering their morphological similarities.

Lu et al. (2017) described similar paired projections on sternum VII in the female of *Pseudobabinskaia martinsnetoi* (Lu, Zhang & Liu, 2017). The segmentation of abdomen cannot be clearly observed due to preservation condition in the holotype female of *P. martinsnetoi*. However, based on the position of these projections in respect of whole abdomen, we consider that these projections actually belong to the sternum VI in *P. martinsnetoi* and are homologous with that in *E. burmana*. Thus, *Electrobabinskaia* and *Pseudobabinskaia* might have close phylogenetic relationship by sharing this feature that is apparently apomorphic. In *P. makarkini* sp. n. a specialised female sternum VI is not present, suggesting that this female trait is not a diagnostic character of the whole family but only for some genera of Babinskaiidae.

The female genitalia of Babinskaiidae consist of paired gonocoxite VIII, gonapophysis VIII (at least present in *E. burmana*), paired gonocoxite IX, paired ectoprocts with well-developed callus cerci, and subanale (at least present in *E. burmana*) (see Fig. 7). The paired digitiform lobes in *Pseudobabinskaia*, interpreted as gonocoxites IX in Lu et al. (2017), are verified to be gonocoxites VIII. This feature appears to be similar to that in some antlion genera (e.g., *Nedroledon* Navás, 1914; see Aspöck and Aspöck 2008: fig. 149) although it is likely convergently derived in Babinskaiidae and Myrmeleontidae. In *Electrobabinskaia* and *Parababinskaia*, the female gonocoxite VIII are less modified.

The presence of subanale (or cataprocessus in New 1981) in Babinskaiidae is also noteworthy. The subanale is a small singular sclerite usually present beneath anus. In Myrmeleontoidea sensu Engel et al. (2018) it is previously reported only in Nymphidae (New 1981). Whether the presence of subanale is apomorphic or plesiomorphic is unknown as it is also present in Psychopsidae (New 1988; Bakkes et al. 2017), a phylogenetically basal family in Myrmeleontiformia that comprises Myrmeleotoidea and Psychopsoidea (Engel et al. 2018).

The phylogenetic position of Babinskaiidae appears to be perplexing with mixture of character states that are shared with Nymphidae, Psychopsidae or Myrmeleontidae. Although Makarkin et al. (2017) deemed the sister group relationship between Babinskaiidae and Nymphidae, Engel et al. (2018) placed Babinskaiidae in a crown group within Myrmeleontoidea, together with Ascalaphidae and Mymeleontidae. In addition, it should be mentioned that the phylogenetic position of Nymphidae is still controversial. Many phylogenetic studies (Winterton et al. 2010; Wang et al. 2017) suggest the basal most position of Nymphidae in Myrmelontoidea sensu Engel et al. (2018). However, a phylogenetic study based on anchored hybrid enrichment data (Winterton et al. 2018) assigned Nymphidae to be the sister group of Ithonidae that is traditionally considered not to be the member of Myrmeleontiformia. Our new finding provides more knowledge on the morphology of Babinskaiidae. However, phylogenetic analysis combining fossil and extant families of Myrmeleontiformia is required in future studies to further elucidate the phylogenetic position of this enigmatic extinct lacewing family.

## Supplementary Material

XML Treatment for Parababinskaia

XML Treatment for Parababinskaia makarkini

XML Treatment for Electrobabinskaia
